# Entropy Theory and Glass Transition: A Test by Monte Carlo Simulation

**DOI:** 10.6028/jres.102.012

**Published:** 1997

**Authors:** J. Baschnagel, M. Wolfgardt, W. Paul, K. Binder

**Affiliations:** Institut für Physik, Johannes-Gutenberg Universität, 55099 Mainz, Germany

**Keywords:** Adam-Gibbs theory, bond-fluctuation model, chemical potential, diffusion coefficient, entropy measurement, Gibbs-DiMarzio theory, glass transition, Monte Carlo simulation

## Abstract

This article reviews the results of a test of the Gibbs-DiMarzio theory by Monte Carlo Simulation. The simulation employed the bond-fluctuation model on a simple cubic lattice. This model incorporates two kinds of interactions: the excluded volume interaction among all monomers of the melt and an internal energy of the chains, which favors large bonds and makes the chains stiffen with decreasing temperature. The stiffening of the chains leads to an increase of their volume requirements, which competes with the packing constraints at low temperatures. This competition strongly slows down the structural relaxation of the melt and induces the glassy behavior. The model therefore takes into account the main opposing forces which the Gibbs-DiMarzio theory makes responsible for the glass transition. For this model the entropy was calculated from the internal and the free energy (derived from the chemical potential and the single chain partition function) and compared with various theoretical predictions: the Gibbs-DiMarzio theory, a theory by Flory for semiflexible polymers and an extended theory by Wittmann considering Milchev’s criticism on Flory’s calculation. The latter extended theory provides the best description of the simulation data.

## 1. Introduction

In 1956 Julian H. Gibbs wrote a seminal paper [1], in which he suggested that a recent theory by Flory [2] could be applied to explain the glass transition of polymer melts. In his study Flory obtained a *negative configurational entropy* for densely packed polymers of sufficient length if their stiffness exceeded a critical value at low temperatures. Flory avoided this paradox by invoking crystallization, whereas Gibbs pointed out that most polymers due to their structural complexity rather undergo a glass transition. Therefore he interpreted Flory’s result as a possible explanation of the Kauzmann paradox [3–5], i.e., of the experimental observation that the configurational entropy difference between the (supercooled) liquid and the crystalline state seems to vanish at a *finite temperature* in the limit of an infinitely slow cooling process. An extensive recent survey of experimental data [6,7] suggests that the extrapolated temperature of the vanishing entropy difference, the Kauzmann temperature *T*_K_ (< *T*_g_), roughly coincides with the Vogel-Fulcher temperature *T*_0_ resulting from fitting the Vogel-Fulcher equation [see Eq. (1)] to pertinent relaxation times or to the viscosity.

This interpretation of Gibbs motivated the development of the Gibbs-DiMarzio theory [8,9], which views the experimental glass transition as a signature of an underlying thermodynamic (second-order) phase transition. This phase transition occurs at *T*_K_ and is driven by the conflict between the bulky low-energy polymer conformations (increasing stiffness) and the contraction of the sample’s volume at low temperatures. At high temperatures the polymers should be able to easily reconcile these opposing forces so that the entropy per monomer *s* is larger than zero. When the temperature is reduced the mentioned conflict gradually starts to develop and decreases the entropy until the number of accessible configurations has become so small that *s* vanishes in the thermodynamic limit. The corresponding state of the melt is called the “ground state of amorphous packing,” in which the melt remains for *T*<*T*_K_.

From this general theoretical treatment many specific predictions have been derived and tested in experiments, such as the variation of the glass transition temperature *T*_g_ (assuming that *T*_g_ behaves as *T*_K_) with pressure [10] or with molecular weight [8,11–14], the temperature dependence of the specific heat at *T*_g_ [15,16], the influence of crosslinks [17–19] and plasticizer [20] on the glassy behavior of the melt, the composition dependence of *T*_g_ for copolymers and polymer mixtures [21], etc. These studies show that the Gibbs-DiMarzio theory provides a rather good description of the experimental situation.

However, to the best of our knowledge, in all tests of the theory only derived quantities were used. No attempt has been made to compare the temperature dependence of the basic quantity, the configurational entropy (which is admittedly hard to measure), with experimental data. With the present work we want to present such test for a computer model of a glassy polymer melt.

This model and some of its properties are discussed in the Sec. 2. Section 3 then gives an outline of the method used to determine the entropy, whereas the Sec. 4 exposes those aspects of the Gibbs-DiMarzio and of related theories, which are necessary for the subsequent comparison. This comparison is discussed in Sec. 5, and finally Sec. 6 contains our conclusions.

## 2. Definition and Properties of the Model

### 2.1 The Bond-Fluctuation Model for Glassy Polymer Melts

**Fig. 1:**
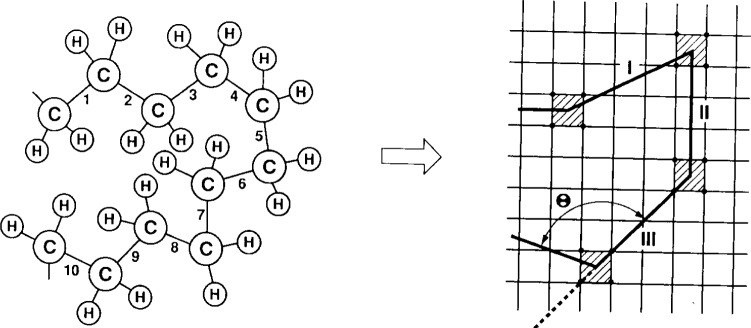
Schematic illustration of the construction of a coarse-grained model for a macromolecule such as polyethylene. In the example shown here, the subchain formed by the three C–C bonds labeled 1,2,3 is represented by the effective bond labeled as I, the subchain formed by the three bonds 4,5,6 is represented by the effective bond labeled as II, etc. In the bond-fluctuation model the length *b* of the effective bond is allowed to fluctuate in a certain range *b*_min_ ⩽ *b* ⩽ *b*_max_, and excluded-volume interactions are modeled by assuming that each bond occupies a plaquette (or cube) of 4 (8) neighboring lattice sites which then are all blocked for further occupation. From Ref. [27].

The bond-fluctuation model [22] was proposed as an intermediate description between a highly flexible continuum treatment and the traditional lattice models, where the flexibility of the chains, described as random or self-avoiding walks [23], is fully determined by the structure of the underlying lattice. It shares with the latter models the simple—and from a computational point of view highly efficient [24]—lattice structure, but distinguishes itself from them by exhibiting a multitude of bond vectors, as also present in continuum models. Due to the lattice structure a monomer of the model does not directly correspond to a chemical monomer, but rather to a group of chemical monomers (typically, such a group comprises 3–5 monomers for simple polymers, such as polyethylene; see Fig. 1 [25]). Since a lattice bond should thus be interpreted as the vector joining the mass centers of these groups, its length and direction will fluctuate. The bond-fluctuation model maps this idea onto a square or simple cubic lattice by associating a monomer with a unit cell of the respective lattice. In order to impose local self-avoidance of the monomers (excluded volume interaction) and to guarantee uncrossability of the bond vectors during the course of the simulation (no phantom chains) the allowed bond vectors are obtained from the sets {(2,0,0), (2,1,0), (2,1,1), (2,2,1), (3,0,0), (3,1,0)} by all symmetry operations of the lattice. This yields 108 different bond vectors.

**Fig. 2:**
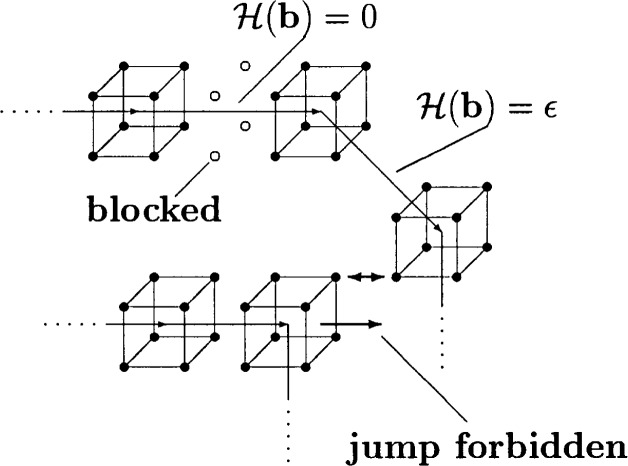
Sketch of a possible configuration of monomers belonging to different chains in the melt in order to illustrate the effect of the model’s energy function and the concept of geometric frustration. All bond vectors shown in this picture have the energy ϵ except the vector (3,0,0) which is in the ground state. This vector blocks four lattice sites (marked by o) which are no longer available to other monomers, since two monomers may not overlap. Due to the excluded volume interaction the jump in direction of the large arrow is also forbidden. This leads to geometric frustration. From Ref. [27].

In addition to the excluded volume interaction an energy function ℋ(***b***) is associated with each bond vector *b*, which favors bonds of length *b* = 3 and directions along the lattice axes (i.e., ℋ(***b***) = 0) in comparison to all other available bond vectors (i.e., ℋ(***b***) = *ϵ*) [26–28]. Figure 2 illustrates the effect of this energy function. When the temperature decreases each bond tries to reach the ground state (i.e., a bond with ℋ(***b***) = 0) and thereby blocks four lattice sites for other monomers. This loss of available volume generates a competition between the energetically driven expansion of a bond and the packing constraints of the melt. Due to this competition some bonds are forced to remain in the excited state (see Fig. 2). They are *geometrically frustrated* [26–28]. The development of the geometric frustration during the cooling process causes the glassy behavior of the model and corresponds to the driving force which the Gibbs-DiMarzio theory makes responsible for the glass transition of polymer melts [8].

For the present study a chain length of *N* = 10 was used. If one takes into account that a lattice monomer roughly corresponds to a group of three to five chemical monomers (see above) our simulation deals with fairly short, oligomeric chains. The cubic simulation box (of linear dimension *L* = 30) contained *K* = 180 chains so that the volume fraction of occupied lattice sites is $\phi =8\;N\;K/L^{3}=0.5\bar{3}$. This value is a compromise between two requirements: it is high enough for the model to exhibit the typical behavior of dense melts at high temperatures [24] as well as pronounced frustration effects at low temperatures [26–28] and low enough to allow for a sufficient acceptance rate of monomer (or chain) moves to make the equilibration of the melt in the interesting temperature range possible [29]. In order to improve the statistics 16 independent simulation boxes were treated in parallel. Thus the total statistical effort involves 28800 monomers, which ensures a high statistical accuracy of the results.

Due to the above mentioned development of the geometric frustration with decreasing temperature the structural relaxation time strongly increases if the usual bond-fluctuation dynamics is used. This makes the equilibration of the melt very time consuming at low temperatures. The usual bond-fluctuation dynamics consists in moving a randomly chosen monomer in a random direction along the lattice axes. These *local* moves are supposed to mimic a random force exerted on a monomer by its environment. They lead to Rouse-like dynamics which is typical of a polymer in a dense melt [30]. However, since we are only interested in equilibrium properties of the melt, this *realistic* dynamics may be replaced by an *artificial* one which uses *nonlocal* moves to speed up the equilibration. A nonlocal move involves a collective motion of all monomers of a chain. Such a collective motion may be realized by the so-called *slithering-snake dynamics* [23,31], for instance. In the slithering-snake dynamics one attempts to attach a randomly chosen bond vector (from the set of allowed bond vectors) to one of the ends of a polymer (both also randomly chosen). If the attempt does not violate the excluded volume restriction, the move is accepted with probability exp [−Δ*E*/*k*_B_*T*], where Δ*E* is the energy difference between the newly added bond and the last bond of the other end of the chain. This last bond is removed, provided the move is accepted. By means of the probability exp [−Δ*E*/*k*_B_*T*] (Metropolis criterion [32]) temperature is introduced into the simulation [33].

### 2.2 A Brief Review of the Model’s Properties

The above mentioned competition between the internal energy and the packing constraints of the polymers prevents the melt from crystallizing so that it may easily be supercooled. During the supercooling process the chains stiffen [26,27,29] and the structural relaxation time of the melt increases [26,27].

**Fig. 3:**
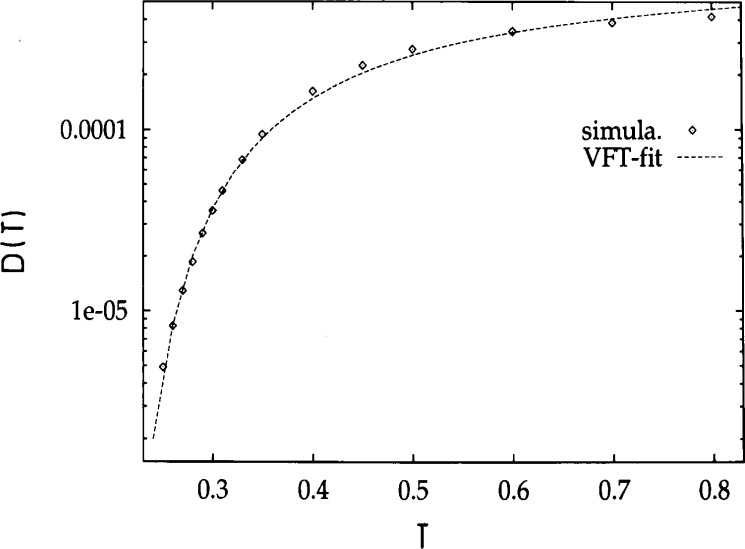
Plot of the diffusion coefficient versus temperature. The dashed line corresponds to a fit by the Vogel-Fulcher equation [Eq. (1)]. From Ref. [27].

This increase of the structural relaxation time is exemplified in Fig. 3 which shows the temperature dependence of the polymer diffusion coefficient [27]. In the available temperature interval the diffusion coefficient decreases by about two orders of magnitude and its temperature dependence may be rather well fitted by a Vogel-Fulcher equation

$$D(T)=D_{\infty }\exp\left[-\frac{C}{T-T_{0}}\right],$$ (1)

yielding *D*_∞_ = 8.61×10^−4^ ± 0.32×10^−4^, *C* = 0.396 ± 0.041 and *T*_0_ = 0.17 ± 0.02. However, the resulting Vogel-Fulcher temperature *T*_0_ ≈ 0.17 certainly overestimates the absolute freezing temperature of the model considerably, since the extrapolation is based on a variation of the diffusion coefficient of two decades only, and one knows from experiments [34–36] where ten decades are available that the fitted values of this Vogel-Fulcher temperature depends on the dynamic range available. The extrapolated result for the Vogel-Fulcher temperature should therefore not be looked upon as an accurate value for the absolute freezing point of the studied model, but rather as an estimate for the crossover temperature to the regime in which the interesting glass physics of this model occurs.

Due to the strong slowing down of the mobility of the polymers the melt starts to vitrify as soon as its structural relaxation time matches the time scale of the simulation. The resulting (kinetic) glass transition temperature depends on both cooling rate [26,27] and chain length [26,37]. It varies with the inverse chain length in a linear and with the cooling rate in a nonlinear fashion. Both of these results are also found in experiments [4,38].

**Fig. 4:**
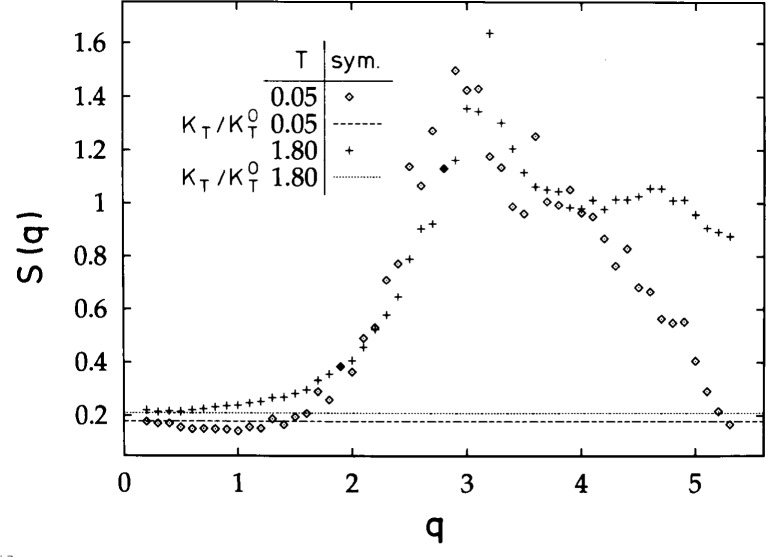
Plot of the collective static structure factor *S* (*q*) vs *q* for *T* = 0.05 (◇; glassy phase) and *T* = 1.8 (+; liquid phase). The cooling rate used in the simulation was *Γ*_Q_ = 4×10^−6^. Additionally, a dashed and a dotted horizontal line are shown which are the isothermal compressibilities $\kappa_{T}/\kappa_{T}^{{}^{\circ}}=T\rho \kappa_{T}$ at the respective temperatures. From Ref. [28].

During the vitrification process the melt maintains its amorphous structure. This is illustrated in Fig. 4 which depicts the wave number (*q*−) dependence of the collective structure factor of the melt at two representative temperatures, one characteristic of the equilibrium liquid state (*T* = 1.8) and one characteristic of the frozen glassy state (*T* = 0.05) [26,28]. The structure factor exhibits the shape expected for an amorphous material. It is small at small *q*-values (where it coincides with the isothermal compressibility), then increases and develops a maximum (the so-called “amorphous halo” [39]) at the typical distance between neighboring monomers (2–3 lattice constants) before it decreases again. This behavior remains unchanged with decreasing temperature. The strong temperature dependence for *q* > 3.7 should not be interpreted physically, since these large *q*-values probe the length scale of a lattice constant, where artifacts due to the underlying lattice must become visible. The important feature of the structure factor is the variation at small and intermediate *q*-values, where it reproduces well-known experimental results [39].

If one further equilibrates the melt in the temperature region close to the kinetic glass transition the model exhibits the phenomenon of physical aging [4,40]. A first analysis of these effects for the present model [41] shows that the approach towards equilibrium is an extremely stretched process which obeys an aging-time-temperature superposition property. Similar results are also found in experiments [4,40].

If one removes these nonequilibrium effects sufficiently the incoherent intermediate scattering function decays in two steps [42,43]. This two-step relaxation behavior is predicted by the mode-coupling approach [43–45] to the structural glass transition and can be described quantitatively by the theory. For the critical temperature of the theory this analysis yields a value of *T*_c_ ≈ 0.15. Since experimentally *T*_c_ > *T*_0_ [43], this result is another evidence that the above shown Vogel-Fulcher fit overestimates the absolute freezing temperature of the model. This conclusion is also corroborated by a recent extensive analysis of many different dynamic properties (relaxation function of the bond vectors, of the radius of gyration, etc., mean-square displacements of the monomers and chains, Rouse-mode analysis), which were calculated from completely equilibrated configurations in an extended temperature regime close to the previously determined *T*_0_ [46]. This study yields *T*_0_ ≈ 0.12−0.13 < *T*_c_, as in experiments.

This keyword-like summary of important properties of the model should illustrate that despite its simplicity the model reproduces experimental findings of (fragile [6]) structural glass formers. Therefore it is a good working model to determine the entropy and to compare the results with the predictions of the Gibbs-DiMarzio theory.

## 3. Entropy and Monte Carlo Simulation

This section briefly describes the main steps and quantities, which are used to determine the entropy by Monte Carlo simulation. To this end, consider a (simple cubic) lattice with *M* sites and *K* polymers. Then the monomer density *ρ* is given by *ρ* = *N K*/*M*. In order to calculate the entropy density *s* (*ρ*, *β*) one has to know the energy and the free energy density as a function of *ρ* and reciprocal temperature *β*, since

s(ρ,β)=βe(ρ,β)−βf(ρ,β) (2)

Whereas the energy density can easily be measured in the simulation as an average of the model’s energy function (see Sec. 2), the free energy density is more difficult to obtain. It may be expressed as [47,51]

$$\begin{array}{c} \beta f(\rho ,\beta )=\frac{\rho }{N}\left[\ln\frac{\rho }{N}-1-\ln\frac{F_{p}(N,\beta )}{N}\right]+\\ \frac{1}{N}\int_{0}^{\rho }d\rho \prime \;\mu_{\text{ex}}(\rho \prime ,\beta ), \end{array}$$ (3)

where ℱ_p_ is the partition function of a single polymer and *μ*_ex_ is the excess chemical potential.

In order to calculate ℱ_p_ we used a method proposed by Kumar et al. [48], whose basic idea is as follows: Imagine a chain of (*N*−1) monomers, to which an additional bond vector shall be attached. This bond vector ***b****_N_*_−1_ experiences a potential *V* (***b****_n_*_−1_) which determines the addition probability *p* (*b_N_*_−1_) by

$$\frac{F_{p}(N,\beta )}{F_{p}(N-1,\beta )}=\sum_{b_{N-1}=1}^{108}\langle \exp[-\beta V(b_{N-1})]\rangle =108p(b_{N-1}).$$ (4)

Iteration of this equation yields

$$F_{p}(N,\beta )=M\;108^{N-1}p(b_{1})\ldots p(b_{N-1}).$$ (5)

Therefore the desired partition function may be expressed in terms of addition probabilities for successive bonds which are accessible in the simulation [47,49,50].

The second input quantity for the calculation of the free energy is the excess chemical potential. Physically the chemical potential is the change in free energy if a test chain (i.e., an additional chain) is added to a polymer melt. When inserted in the melt (m) the test chain (t) experiences a potential *V*_mt_ with the surrounding polymers, the Boltzmann factor of which, averaged over all melt and test chain configurations, determines the insertion probability *p*_ins_ and the excess chemical potential by [51]

$$\beta \mu_{\text{ex}}(\rho ,\beta )=-\ln\langle \exp[-\beta \;V_{\text{mt}}]\rangle =-\ln p_{\text{ins}}(\rho ,\beta ).$$ (6)

Quite generally, the insertion probability for polymers is a very small quantity. For instance, for the present model it is *p*_ins_ ≈ 10^−11^ already at infinite temperature and decreases still further with falling temperature [47]. The reason for these small values is that a randomly chosen test chain configuration, inserted at random in the melt, is likely to overlap many times with the surrounding polymers. Since every single overlap forbids the insertion, successful attempts stem from the events that the chosen configuration happens to fit in the cavity of the melt at the point of insertion. Since these events are very seldom, the random insertion method, which can successfully be applied to small molecules, is very inefficient for polymers. In order to circumvent this inefficiency various methods have been designed [49–51]. We use a method proposed by Müller et al. [49,50], whose basic idea is as follows: Instead of strictly forbidding overlaps between the test chain and the melt every overlap is penalized by a finite potential

$$-\beta \;V_{\text{mt}}(N_{0},\lambda )=N_{0}(x_{\text{mt}})\ln\lambda ,$$ (7)

where *N*_0_(*x*_mt_) is the number of overlaps, which depends on the melt and the test chain configuration *x*_mt_, and *λ* is a tunable parameter ranging between 0 and 1. The value *λ* = 0 corresponds to complete excluded volume interaction, whereas *λ* = 1 means no excluded volume interaction. The intermediate *λ*-values between these two extreme states are used as auxiliary variables in the (extended-ensemble) simulation in order to gradually approach the desired case of complete excluded volume interaction and to gather sufficient statistics for it.

## 4. Entropy Approximations: Gibbs-DiMarzio and Related Theories

In the following section the simulation results for the entropy will be compared with the prediction of the Gibbs-DiMarzio theory [8], of a theory by Flory [2] and of a theory by Wittmann [52] which considers Milchev’s criticism on Flory’s calculation [53]. The present section is devoted to introduce those aspects of these theories which are needed for the comparison.

Consider a lattice with *M* sites and *K* polymers of length *N*. Each monomer is supposed to occupy a single lattice site (contrary to the bond-fluctuation model; see Sec. 2). Furthermore, let *Ω* denote the total number of configurations of the melt. All of the above mentioned theoretical approaches factorize *Ω* into an intra- and an interchain contribution so that the entropy density is approximated by

$$s=\frac{1}{M}\;\ln\;\Omega \approx \frac{1}{M}\;\ln\;[\Omega_{\text{intra}}\Omega_{\text{inter}}].$$ (8)

The intrachain contribution accounts for the increase of the chain’s stiffness during supercooling. This is modeled in the theories by associating an energy with the bond angle. The energy is 0 if two successive bonds are colinear and *ε* otherwise. For such a *two-level* system the probability that two successive bonds are not colinear (“flexed”), the so-called *flexibility f*, reads

$$f=\frac{\left(z-2)\exp[-\beta \varepsilon \right]}{1+(z-2)\exp[-\beta \varepsilon ]},$$ (9)

where *z* is the coordination number of the lattice. With this assumption about the internal energy of a polymer the intrachain contribution to *Ω* is given by the following binomial distribution [52]

$$\Omega_{\text{intra}}=\left(\begin{array}{l} K(N-2)\\ fK(N-2) \end{array}\right){\left(\frac{1}{z-1}\right)}^{\left(1-f)K(N-2\right)}{\left(\frac{z-2}{z-1}\right)}^{fK(N-2)}.$$ (10)

In addition to *Ω*_intra_ an expression is needed for the number of different ways in which *K* polymers may be placed onto the lattice. This interchain contribution to *Ω* is given by

$$\Omega_{\text{inter}}=\frac{1}{2^{K}}\frac{1}{K!}\prod_{k=0}^{K-1}v_{k+1}.$$ (11)

It involves the total number of configurations *v_k_*_+1_ that the (*k*+1)th chain may adopt if there are already *k* chains on the lattice. Approximately, *v_k_*_+1_ consists of three factors

$$v_{k+1}\approx (M-kN)\times N_{\text{empty}}^{N-1}\times z{\left(z-1\right)}^{N-2}.$$ (12)

The first factor is the number of remaining empty lattice sites and thus of potential starting points for the (*k*+1)th polymer, whereas the last factor represents the number of possibilities to place the remaining (*N*−1) monomers onto the lattice if immediate backfolding is forbidden. The second factor approximately accounts for the self- and mutual avoidance of the polymers. It is related to the probability that the newly chosen site for the next monomer of chain (*k*+1) is in fact empty. The three theories mentioned above differ from each other in the ansatz made for *N*_empty_. Flory uses the volume fraction of empty lattice sites for *N*_empty_, i.e.,

$$N_{\text{empty,F}}=1-\frac{kN}{M},$$ (13)

whereas Gibbs and DiMarzio and Milchev work with

$$N_{\text{empty,}\;\text{GDM}}=\frac{1-kN/M}{1-k(N-1)/(Mz/2)}$$ (14)

and

$$N_{\text{empty,}\;M}=\frac{1-kN/M}{1-k/K}=\frac{M-kN}{M-kM/K},$$ (15)

respectively. They divide Flory’s ansatz by expressions which approximately take into account that not all empty lattice sites can serve as starting points for the new polymer, but only those which lie outside of the volume already consumed by the other *k* chains. To estimate this volume Gibbs and DiMarzio argue that there are *Mz*/2 different nearest neighbors (on a lattice with periodic boundary conditions) so that (*N*−1)/(*Mz*/2) is the volume fraction occupied by a chain, whereas Milchev assumes that a chain consumes on average *M*/*K* sites.

With these different ansatzes the entropy density can be calculated. The final results read [52]

$$s_{F}(f,K,N,H,z)=s_{M}(f,K,N,H,z)-\frac{KN}{KN+H}\left[1-\frac{1}{N}\right]$$ (16)

in Flory’s approximation and

$$s_{\text{GDM}}(f,K,N,H,z)=s_{M}(f,K,N,H,z)+\frac{z(K\tilde{N}+H)}{2(KN+H)}\ln\frac{\left(K\tilde{N}+H\right)}{\left(KN+H\right)}$$ (17)

for the Gibbs-DiMarzio theory. In Eqs. (16) and (17) *s*_M_ is the results of Milchev’s theory, *H* denotes the number of holes (i.e., of empty lattice sites: *H* = *M*−*KN*) and $\tilde{N}$, is number of nearest neighbors of a (rodlike) polymer [52]. In the limit of the completely filled lattice (*H* → 0), of *N* → ∞ and *T* → 0 (i.e., *f* → 0), Milchev’s entropy vanishes [52] so that

$$s_{F}(f,K,N,H,z)\overset{H,f\to 0,N\to \infty }{\to }-1$$ (18)

and

$$\begin{array}{c} s_{\text{GDM}}(f,K,N,H,z)\overset{H,f\to 0,N\to \infty }{\to }\frac{z}{2}\\ \times \left[1-\frac{2}{z}\right]\ln\left[1-\frac{2}{z}\right]\overset{z\to \infty }{\to }-1. \end{array}$$ (19)

The negative entropy of Eq. (19) in the low temperature limit is interpreted as the theoretical counterpart of the Kauzmann paradox [3–5]. The Gibbs-DiMarzio theory resolves this paradox by associating a second-order phase transition from an ideal glass to a liquid phase with the temperature *T*_K_ at which the configurational entropy vanishes. Such a transition does not occur in Milchev’s theory, where the entropy remains positive for any finite temperature [52]. This means that the choices made in Eqs. (13)–(15) do not only introduce quantitative corrections to the final result, but lead to a qualitatively different behavior. It is therefore interesting to test which of the theoretical approaches describes the simulation data better.

## 5. Comparison of Theory and Simulation

### 5.1 Determination of Input Parameters

In order to perform a comparison between the above outlined theories and the simulation data the relevant input parameters, the flexibility *f*, the number of holes *H* and the coordination number *z* of the lattice, have to be determined for the bond-fluctuation model.

**Fig. 5:**
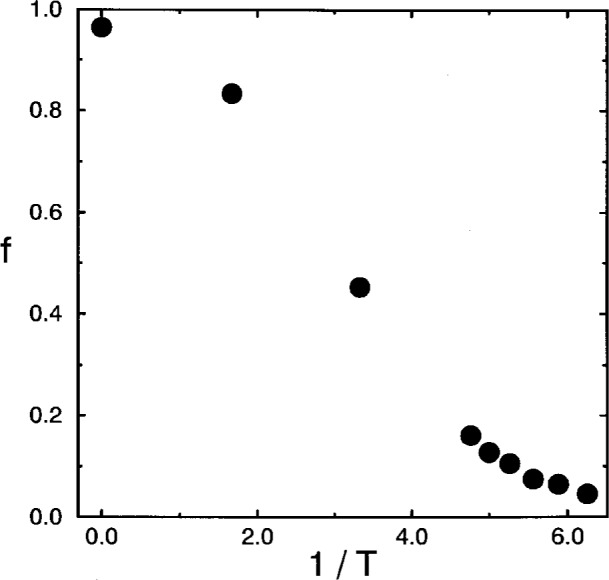
Fraction *f* of bonds in the excited state versus inverse temperature. From Ref. [54].

The theories associate a two-level system with the bond angle, whereas the present model uses a two-level system for the bond length. Therefore we replace the flexibility by the fraction of bonds in the excited state. The temperature dependence of the so-defined *f*-parameter is shown in Fig. 5 [54]. At high temperatures almost all bonds are in the excited state. As the temperature decreases more and more bonds enter the expanded ground state (see Fig. 2) and the *f* -parameter decreases. In addition, the expansion on the length scale of a bond also induces an expansion of the chains as a whole, which entails an increase of the chain’s stiffness [29]. This is exactly the effect that the theories try to model by the introduction of the flexibility. Note that simulations using two-level systems associated with the bond angle (as in the analytic theories) give evidence for the formation of strong nematic-type short-range order among the chains, which is not typical of the glass transition of most polymers, and hence such models are less suitable for testing the entropy theory of the glass transition. The second input quantity is the number of holes. In order to determine *H* remember that a monomer of the bond-fluctuation model does not correspond to a single lattice point (as in the theories), but to a whole unit cell of the simple cubic lattice. Therefore a hole should be interpreted as an empty unit cell. With this identification one can define an effective density *ρ*_eff_ by [54]

$$\rho_{\text{eff}}=\frac{KN}{KN+H(T)}.$$ (20)

**Fig. 6:**
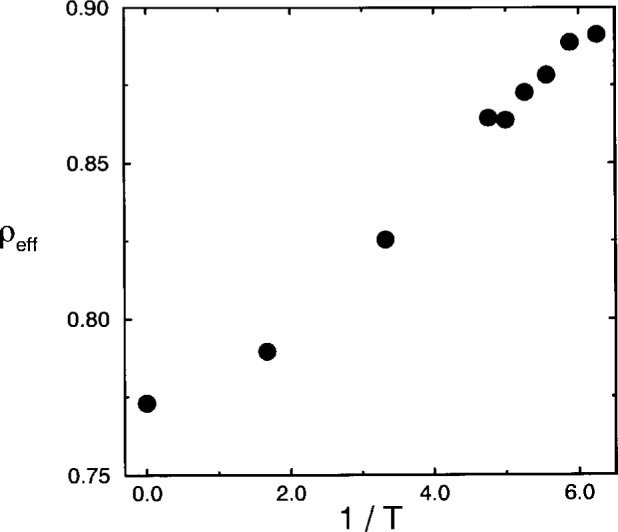
Effective density *ρ*_eff_ versus inverse temperature. From Ref. [54].

The temperature dependence of this density is shown in Fig. 6 [54]. For all temperatures *ρ*_eff_ is larger than the volume fraction of occupied lattice sites $\phi =0.5\bar{3}$, which illustrates that the melt is in fact much denser than could have been anticipated on the basis of *ϕ* alone. Furthermore, the effective density increases with decreasing temperature, since the accompanying increase of the number of bonds in the ground state reduces the available volume and thus makes the density rise.

**Fig. 7:**
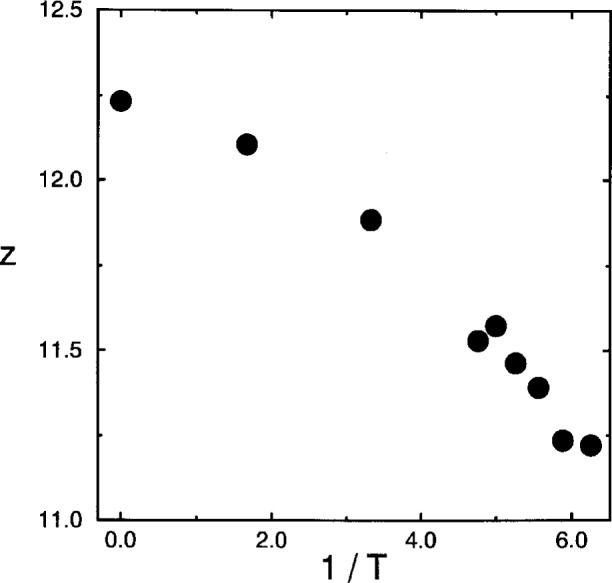
Coordination number *z* versus temperature. From Ref. [54].

Finally, the coordination number *z* of the lattice has to be determined. In the theories, *z* coincides with the number of nearest neighbors. Therefore a natural choice is to take *z* as the number of monomers in a sphere with radius $b_{\max}=\sqrt{10}$ (see Sec. 2) around a central monomer. Figure 7 shows that the resulting *z*-values range between 11 and 12, which is typical of dense (amorphous) packing and that *z* decreases with decreasing temperature due to the expansion of the bond vectors [54].

### 5.2 Results

**Fig. 8:**
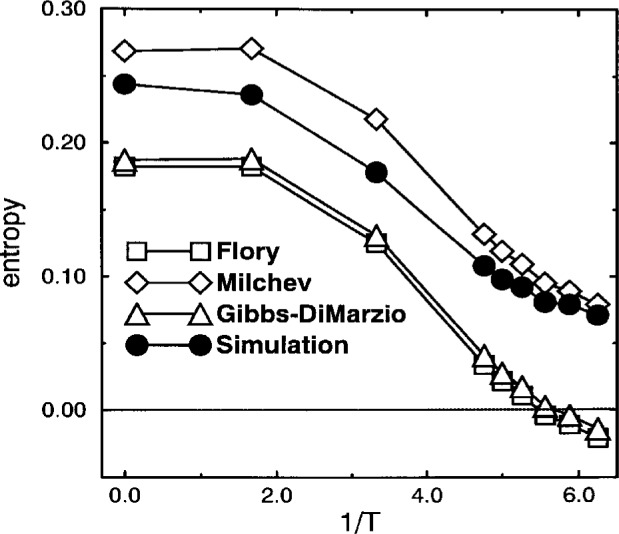
Comparison of the temperature dependence of the entropy per lattice site *s* with the Milchev, Flory, and Gibbs-DiMarzio theory [see Eqs. (16)–(17). From Ref. [54].

Having made the above discussed identifications, *f*, *H* and *z* can be inserted in Eqs. (16)–(17). The resulting temperature dependence is compared with that of the simulation data in Fig. 8 [54]. As expected, the simulated entropy decreases with decreasing temperature. However, instead of exhibiting a clear tendency to vanish at a finite temperature its decrease gradually weakens with falling temperature so that it rather seems to stay larger than zero also at very low temperatures, where no further simulation points are up to now available. Therefore Milchev’s theory lies closer to the simulation data than the Gibbs-DiMarzio or the Flory theory.

**Fig. 9:**
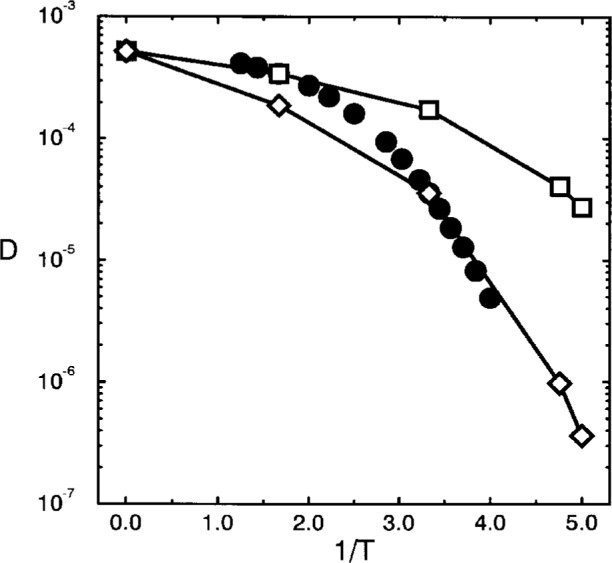
Comparison of the temperature dependence of the diffusion coefficient with the Adam-Gibbs formula [Eq. (21)]. See text for further details. From Ref. [54].

However, taking into account that *T*_K_ = 0 in Milchev’s theory [52] and that there is the Adam-Gibbs relation [55]

$$D(T)=D_{\infty }\exp\left[-\frac{C}{Ts(T)}\right],$$ (21)

which was proposed as a possible rationalization of the Vogel-Fulcher equation, the found temperature dependence of the entropy challenges the Vogel-Fulcher analysis of the diffusion data (see Fig. 3) [54]. Therefore Fig. 9 directly compares Eq. (21) with the measured diffusion coefficients of the chains. Since there are only two data points for the entropy at *T* = 0.3 and *T* = 0.6 in the temperature interval where *D* is available, the prefactor in Eq. (21) was taken from the simulation (*D*_∞_ = 5.2×10^4^) and the constant *C* was adjusted in such a way that Eq. (21) either coincided at *T* = 0.3 or at *T* = 0.6 with the simulation data. Despite the preliminary status of this comparison the result for *T* = 0.3 is not unreasonable. This suggests that the Adam-Gibbs formula could be a valid description for the temperature dependence of the structural relaxation times of the present model and that the absolute freezing point should be smaller than *T*_0_ ≈ 0.17.

## 6. Concluding Remarks

This paper summarizes the results about the temperature behavior of the entropy for a simple model of a supercooled polymer melt. The model consists of linear monodisperse chains on a (simple cubic) lattice, which interact by a hard core potential and possess an internal energy that increases the chains’ stiffness with decreasing temperature. During supercooling a competition therefore arises between the tendency of a single chain to expand and the corresponding enlarged volume requirement of all chains. These opposing forces strongly slow down the structural relaxation and mimic the mechanism that the Gibbs-DiMarzio theory suggests to be responsible for the glassy behavior of polymer melts. In fact, this suggestion partly inspired the design of the model.

However, when the temperature dependence of the entropy is compared with the theoretical prediction one finds that the shape of the curves are very similar, but that the simulation data are shifted upwards with respect to the theory. A similar shift is also obtained theoretically by Wittmann’s analysis of Milchev’s criticism. Therefore Milchev’s theory approximates the simulation data better than Gibbs-DiMarzio’s or Flory’s theory. This finding leads to several conclusions and questions: (1) Presumably, the Gibbs-DiMarzio theory (as well as the other theories) can well describe the temperature dependence of the model’s specific heat *c*, since *c* = *T*∂*s*/∂*T*, as it is also found in experiments. In order to test this conjecture more data points are needed at low temperatures. This is a prospective topic for future work. (2) Gibbs-DiMarzio’s correction of Flory’s ansatz for *N*_empty_ [see Eq. (14)] only implies minor changes for the entropy. Both theories predict the entropy to vanish at about the same temperature (i.e., *T*_K_ ≈ 0.17 – 0.18). Interestingly, this temperature coincides with the Vogel-Fulcher temperature *T*_0_ obtained from the diffusion coefficient. Whether this is a mere coincidence or whether there is an explanation on the basis of the model’s properties is still unclear. (3) The steepness of the entropy decrease in the simulation becomes weaker with decreasing temperature. This tendency to rather level off than to vanish suggests that the entropy remains larger than zero also at very low temperatures, where no data points are up to now available. Certainly, further simulations are necessary to clarify this behavior. (4) An interesting question in this context is the validity of the Adam-Gibbs relation [see Eq. (21)]. This relation implies for the relaxation time *τ* ∝ exp[*A*/*Ts*], contrary to the usual expectation *τ* ∝ exp[*βf*] ∝ exp[*s*/*k*_B_]. Our simulation only provides a first indication that the Adam-Gibbs relation could be applied. To test this indication the structural relaxation time has to be determined at lower temperatures. Work in this direction is underway [46]. Since Milchev’s theory is closest to the simulation data, it would then also be possible to check its prediction for the temperature dependence of the relaxation time, i.e., [56]

$$\tau (T)\propto \exp\left[{\left(\frac{\theta }{T}\right)}^{\alpha }\right]\;\text{with}\;\alpha =\frac{2c}{z}.$$ (22)

In order to apply this equation an extension of entropy measurement is also needed due to the dependence of the exponent *α* on the specific heat.

## Glossary

*b*: bond vector of length *b*
*c*: specific heat
*D*: constant
*D*_∞_: diffusion coefficient at infinite temperature
*D*(*T*): diffusion coefficient at temperature *T*
*e*(*ρ, β*): energy density as a function of monomer density *ρ* and reciprocal temperature *β*
Δ*E*: energy difference in the Metropolis criterion
*f*(*ρ, β*): free energy density as a function of monomer density *ρ* and reciprocal temperature *β*
*f*: flexibility parameter [see Eq. (9)]
*H*: number of holes (empty lattice sites or unit cells)
ℋ(***b***): bond vector energy function of the bond-fluctuation model
*k*_B_: Boltzmann constant
*K*: number of polymers per simulation cell
*L*: linear dimension of the simulation cell
*M*: total number of lattice sites
*N*: chain length
*N*_0_: number of overlaps between a test chain and the melt [see Eq. (7)]
*N*_empty_: approximate number of adjacent empty lattice sites for placing of a new monomer during the construction of a polymer [see Eq. (12)]
*N*_empty,F_: Flory’s approximation for *N*_empty_ [see Eq. (13)]
*N*_empty,GDM_: Gibbs-DiMarzio’s approximation for *N*_empty_ [see Eq. (14)]
*N*_empty,M_: Milchev’s approximation for *N*_empty_ [see Eq. (15)]
*p*(***b***): addition probability for a bond vector *b* [see Eq. (4)]
*p*_ins_(*ρ*,*β*): insertion probability for a test chain in a melt at monomer density *ρ* and reciprocal temperature *β* [see Eq. (6)]
*q*: wave number
*s*(*ρ*,*β*): entropy density as a function of monomer density *ρ* and reciprocal temperature *β*
*s*_F_: Flory’s approximation for the entropy density [see Eq. (16)]
*s*_GDM_: Gibbs-DiMarzio’s approximation for the entropy [see Eq. (17)]
*s*_M_: Milchev’s approximation for the entropy [see Eqs. (16) and (17)]
*S*(*q*): collective static structure factor
*T*: temperature
*T*_c_: critical temperature of mode-coupling theory
*T*_g_: (kinetic) glass transition temperature
*T*_K_: Kauzmann temperature
*T*_0_: Vogel-Fulcher temperature
*V*(***b****_N_*_−1_): potential experienced by a bond when added to a chain of (*N*−1) monomers [see Eq. (4)]
*V*_mt_: potential between an inserted test chain (t) and the melt (m)
*V*_mt_(*N*_0_,*λ*): soft potential between an inserted test chain (t) and the melt (m) [see Eq. (7)]
*z*: coordination number of the lattice
ℱ_p_: single chain partition function [see Eq. (5)]
*β*: 1/*T*
*ϵ*: energy parameter of ℋ(***b***) (units: *ϵ / k*_B_ = 1)
*ε*: energy parameter of the bond angle in the Gibbs-DiMarzio and the other theories [see Eq. (9)]
θ: effective energy barrier (in temperature units) of the relaxation time [see Eq. (22)]
*κ_T_*: isothermal compressibility
*λ*: excluded volume parameter [see Eq. (7)]
μ_ex_: excess chemical potential [see Eq. (6)]
*v_k_*: number of configurations for the *k*th chain if there are already (*k*−1) chains on the lattice [see Eq. (12)]
*ρ*: monomer density (= *K N/M*)
*ρ*_eff_(*T*): effective monomer density (= *K N/* [*KN* + *H*]) [see Eq. (20)]
*τ*: structural relaxation time
*ϕ*: volume fraction of occupied lattice site in the bond-fluctuation model (= 8*K N*/*L*^3^)
*Ω*: total number of melt configurations [see Eq. (8)]
*Ω*_intra_: intrachain contribution to *V* [see Eq. (10)]
*Ω*_inter_: interchain contribution to *V* [see Eq. (11)]
